# Recurrent Admissions for Diabetic Foot Complications

**DOI:** 10.5704/MOJ.1307.010

**Published:** 2013-07

**Authors:** CL Ang, YJ Lim

**Affiliations:** Department of Orthopaedic Surgery, Changi General Hospital, Singapore; Department of Orthopaedic Surgery, Changi General Hospital, Singapore

## Abstract

Diabetic foot complications are a significant source of morbidity and mortality. Patients who undergo recurrent admissions for the same diabetic foot problems represent a difficult subgroup to treat. From July 2007 to June 2008, there were 38 such patients who were admitted recurrently. Eighteen patients (47%) were re-admitted because of previous refusal of surgical treatment. Eighteen patients (47%) received treatment as necessary but were still readmitted for recurrent infection at the same wound site. Assessment of patients’ compliance to outpatient treatment was found to be generally lacking. As a significant proportion were re-admitted because of previous refusal of surgery, a trained counselor may be suitable in counselling patients for debridement or amputation surgery.

**Key Words:**

Diabetic foot, complications, ulcer, abscess, amputation

## Introduction

Diabetes-related foot complications are a major health care problem with significant socioeconomic burden^1^-^3^. They encompass a variety of clinical conditions ranging from simple ulceration to deep abscess collection and wet gangrene. The pathophysiology of these conditions usually involves a combination of vascular stenosis, tissue ischemia, infection, and peripheral neuropathy^4^. These conditions often require a multidisciplinary approach with involvement of various auxiliary health support services.

The primary aim of treatment of diabetic foot problems is limb preservation. The risk of lower limb amputation in diabetic patients has been estimated at 8 to 12.3 times the risk in non-diabetic patients^5^-^6^. However, reviews of inpatients undergoing treatment for diabetic foot problems have shown significant variability in clinical assessment, investigation and management^7^-^9^. The authors of these reviews have suggested that there is significant room for improvement in the assessment and management of acute diabetes-related foot complications.

In our clinical experience, a significant number of patients are admitted repeatedly for treatment of diabetes-related foot complications, and not infrequently it is for treatment of a persistent problem which has previously already received appropriate medical care. We asked ourselves: what was the management of these patients in their index admissions and what were the factors that led to their re-admission? Anecdotally practitioners are well aware of reasons including non-compliance to treatment or refusal of treatment that would lead to re-admissions, but the current English literature is lacking in the description of this category of patients. To gain preliminary insight into our questions, we conducted a retrospective review of patients with diabetic foot problems who were admitted to our institution for treatment and subsequently re-admitted for the same foot conditions. We concentrated on patients who represented with the same foot conditions in order to review our treatment outcomes and elucidate the potential factors that might result in failure of treatment.

## Materials and Methods

This is a descriptive study done via a retrospective review of patients admitted to a public hospital over a period of one year (from July 2007 till June 2008). Approval from the Institutional Review Board was obtained prior to the commencement of the study. In our institution, diabetic patients with foot complications are routinely admitted to the Department of Orthopaedic Surgery. From our department, we will then refer to the vascular surgeon if there is evidence of significant peripheral vascular disease which requires surgical or procedural treatment. A list of patients was obtained from the Medical Information Management Department satisfying the following criteria: admissions to the Department of Orthopaedic Surgery during the study period; a re-admission to the same department within 30 days from the date of discharge; a diabetic-foot related 2009 ICD-9-CM (International Statistical Classification of Diseases) code in the first and subsequent admissions. The ICD codes were chosen to represent diagnoses including diabetes mellitus, ulceration, cellulitis, abscess, gangrene and osteomyelitis. We also included ICD codes where there was a possibility of diabetic foot pathology (Table I) in order to overcome the possible problem of under-reporting of diabetic foot problems.

From the list of patients generated using the ICD codes, case records were screened through to assess the reasons for the primary and subsequent admissions. Only patients admitted for treatment of diabetic foot problems were included. Furthermore, patients were included in the review only if they were re-admitted for treatment of the same diabetic foot problem as the primary admission. The following specific information was then extracted from the case records using a standardized data collection form: Number of re-admission episodes, reasons for each re-admission episode, patients’ demographic data, documentation of clinical examination of the diabetic foot problem, relevant investigation results including inflammatory markers (total white count, erythrocyte sedimentation rate, C-reactive protein), radiographic findings and wound swab cultures, documentation of patients’ understanding of condition and compliance to treatment, assessments by the relevant auxiliary health services (for example: the diabetic nurse educator, the podiatrist, and the dietician)

## Results

Seventy-six patients with 179 re-admission episodes were identified using the selected ICD codes (Table I). We found 38 patients with 57 re-admission episodes for persistent diabetic foot problems. There were 25 males (65.8%) and 13 females (34.2%). The age range was from 30 to 91 years with a mean age of 61.7 years. Racially, the majority of the patients were Malays (21 patients, 55.3%), followed by Chinese (16 patients, 42.1%) and Indian (1 patient, 2.6%). All the patients had type 2 diabetes mellitus. Thirty-five (92.1%) patients had at least one concomitant vascular risk factor (hypertension, smoking, hypercholesterolemia, ischaemic heart disease or cerebrovascular accident) and 28 (73.7%) had at least two other risk factors.

The distribution of the 57 re-admission episodes according to the number of re-admissions and the foot condition leading to re-admissions is shown in Table II and III. There were six patients with evidence of osteomyelitis on radiographs (three patients had ulcers, two had wet gangrene, and one had abscess). Out of the 38 patients, 18 (47.4%) refused surgical treatment or vascular angioplasty in their primary admissions and were advised on the risk of progressive infection. Eighteen patients underwent treatment as deemed medically necessary: 11 (28.9%) underwent surgical treatment and seven (18.4%) were treated conservatively with intravenous antibiotics and wound dressings. Two (5.3%) patients who received conservative treatment were identified clearly with chronic non-compliant behaviour and poor self-motivation. One of the two patients had 10 re-admission episodes and was repeatedly admitted for conservative treatment of a right sole neuropathic ulcer. The treatment consisted of antibiotics and regular assessments by a nurse clinician trained in wound care and dressings. He was noted to have mild mental retardation and poor self motivation with non-compliance to regular outpatient dressings. The other patient was also identified with poor compliance and self-motivation; he had two re-admission episodes within the study period for conservative treatment of a non-healing foot ulcer.

Eighteen patients (47.4%) had refused surgical or vascular interventional treatment in their primary admissions. Of these, nine patients (23.7%) refused surgical debridement or amputation surgery, and seven patients (18.4%) who underwent initial surgical debridement or ray amputations later refused further debridements or more proximal amputations when their wounds remained infected or were not healing well. In addition, two patients (5.3%) refused lower limb angioplasties for critical vessel stenosis of more than 75% on arterial duplex scan. The conditions of these 18 patients comprised of abscesses (four patients, 10.5%), infected ulcers (six patients, 15.8%) and wet gangrene (eight patients, 21.1%). All 18 patients’ case records had a documented discussion of the risks and benefits of the proposed intervention, including possible outcomes if the condition was not treated as recommended. Loss of limb and/or life was consistently documented in the records. However, the exact reasons for refusal were often not specifically identified.

When re-admitted, 10 out of these 18 patients (55.6%) subsequently agreed to surgical treatment including one who agreed to lower limb angioplasty. Seven patients (38.9%) still refused surgical treatment. One patient (5.6%) with chronic osteomyelitis of the toe was eventually treated conservatively with suppressive antibiotics. The seven patients who still refused surgical treatment consisted of five patients who were advised for below knee amputation, one for above knee amputation and one for big toe ray amputation. Two of the patients who refused major limb amputation eventually died during their period of re-admission, and one subsequently died a month later at home.

**Fig 1:**
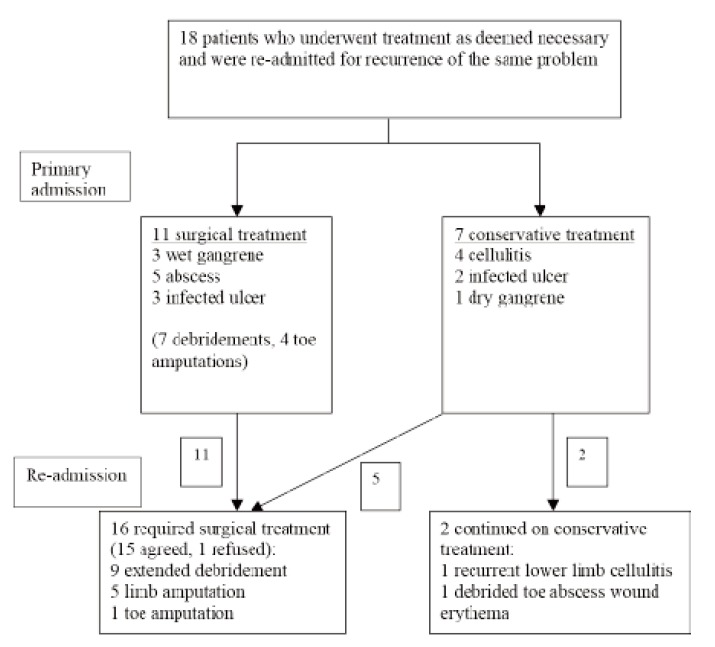
: Treatment pathway of the 18 patients who were treated as necessary but re-admitted.

Eighteen other patients (47.4%) underwent treatment in accordance with medical treatment plan during their primary admissions. This included commencement of appropriate intravenous antibiotics and surgical debridements or amputations as required. Fig. 1 shows the distribution of the 18 patients according to the method of treatment in their primary admissions and the subsequent treatment during their re-admissions. These patients had satisfactory wound conditions during inspection by team doctors upon their discharge from the primary admissions. Sixteen of the patients were re-admitted for recurrent infections of the same wounds and two were re-admitted for development of gangrene. These eighteen patients were re-admitted after a median of 18.2 days (range 1 – 29) compared to a median of 12.0 days (range 1 – 24) for the other 18 patients who refused surgical treatment initially. Review of these 18 patients’ case records showed that there was a paucity of documentation on the patients’ adherence to outpatient dressings or medications. Table V shows the clinical assessments undertaken for the patients in this study and reflected low rates of documentation of adherence to outpatient treatment and patient understanding of their own conditions. The one patient who was re-admitted after one day despite having received treatment was a case of a heel abscess for which surgical drainage was performed. She was treated with intravenous antibiotics postoperatively and her wound was observed for four days. On the day of discharge, the wound was slightly sloughy but there was no pus. The day after discharge, the patient re-presented to the emergency department with a temperature of 38°C and clinical findings of a sloughy wound. She was initially treated conservatively for the first 5 days but the wound remained sloughy and she eventually underwent a repeat surgical debridement.

Upon re-admission, sixteen patients were deemed to have required surgical treatment and two were continued on conservative treatment (Fig. 1). The HbA1c values for this group ranged from 6.2% to 14.1%, with an average of 9.2%. Biochemical markers and haemoglobin levels for all the patients in this study and the group of 18 patients who were treated but yet still re-admitted are shown in Table IV. There was a variable rate of assessment by the para-medical services including the podiatrist, diabetic nurse educator and the dietician (Table V). All patients had basic blood haematological and biochemical tests and almost all had inflammatory markers monitored. Other investigations such as wound swab culture or albumin levels were less frequently done (63.2% and 34.2% respectively).

## Discussion

The findings of this study suggest that a significant proportion of our local patients with diabetic foot-related complications refuse surgical treatment even when advised to by doctors. Many of them subsequently agreed to surgery when their conditions failed to improve with conservative treatment leading to re-admission. Inevitably, re-admission episodes are associated with emotional and financial strains on the patient; they also represent potentially preventable sources of expenditure to the health care system. A smaller group of patients still refused any surgery despite being admitted to hospital again. Our anecdotal experience is that many patients are firmly opposed to the loss of a limb even if it is medically required and life-saving. Due to the religious beliefs of the Muslims, most of the patients would want to be buried whole, and thus they usually refuse amputation. Also, the common sentiment amongst patients is that losing a limb makes one a ‘cripple’, and they would rather lose their lives than a limb. The key to this problem is correcting the patient’s misconceptions about losing a limb and the eventual functional status. However, doctors on busy morning ward rounds often do not adequately help patients overcome the emotional hurdle to accepting an amputation. We postulate that a trained counselor with expertise and experience in amputation counselling will reduce the rate of refusal of surgery. That said, doctors are not absolved of our responsibility in obtaining a complete assessment of such patients, including patients’ compliance to outpatient treatment and their understanding of their own disease conditions.

Eighteen patients (47.4%) in our review underwent medical treatment as deemed necessary in their primary admissions but were still re-admitted for recurrence or progression of infection affecting the same wound or in the areas surrounding the wound. These 18 patients had poor glycaemic control as a group with an average HbA1c reading of 10.0%. Poor glycemic control is known to constitute a significant risk factor towards susceptibility to infection^10^ . A main treatment goal would thus be to improve glycemic control in diabetics to reduce the risk of diabetic foot infections. Inpatient administration of medications and blood glucose monitoring are done strictly under the supervision of trained nursing staff. However, in the outpatient setting, patients’ adherence to dressing changes, self care of foot wounds and compliance to medications often constitute an unknown factor in the contribution to recurrent wound infections. Our study found that documentation of these aspects was generally lacking. Assessing patients’ outpatient care patterns may reveal incorrect practices by patients, thus giving healthcare workers an opportunity to address them. This represents an area where the medical team should aim to improve. We also found considerable variability in inpatient assessment by the various paramedical services, namely the nurse educator, podiatrist and dietician. This lack of consistency in assessment is, unfortunately, similar to the findings in previous studies^7^-^9^, suggesting that this problem may not be unique to any one institution, but rather, represents an area where medical teams everywhere may aim to improve in. A recent publication by Rümenaph et al11 lends support to our recommendation of improving the assessment and management of outpatient care to reduce re-admission rates.

The weaknesses of this descriptive study include the retrospective nature and the lack of complete documentation in many of the patients’ case records. That said, it can be taken as a learning point for ourselves to improve on clinical assessement and documentation. Secondly, being a retrospective descriptive study, there is no opportunity to observe the effects of intervention, for example by having a trained counselor spend time with a patient to counsel in accepting an amputation.

**Table I:**
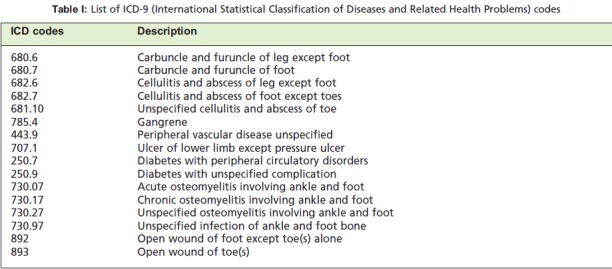
: List of ICD-9 (International Statistical Classification of Diseases and Related Health Problems) codes

**Table II:**
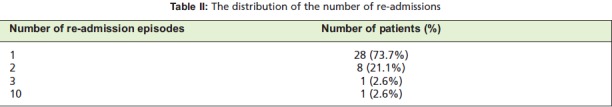
: The distribution of the number of re-admissions

**Table III:**
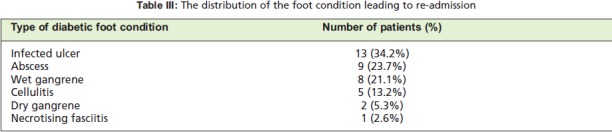
: The distribution of the foot condition leading to re-admission

**Table IV:**
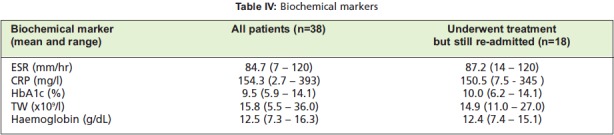
: Biochemical markers

**Table V:**
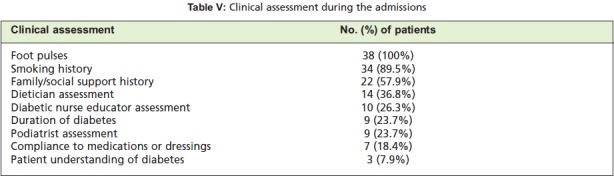
: Clinical assessment during the admissions

## Conclusion

Our study found that a significant proportion of our local patients presenting with diabetic foot complications refuse surgical treatment, leading to re-admission episodes. These re-admission episodes pose a significant socioeconomic burden both on the patients and on the health care system. On the other hand, almost half of the patients in our review were re-admitted for recurrent infection of the same foot problem despite receiving adequate treatment in their primary admissions. Compliance to outpatient medications and wound dressings should be an integral part of assessment of these patients. It is helpful to perform prospective studies where a cohort of diabetic foot patients are monitored beyond their initial hospitalizations to track the success or failure (thereby leading to re-admissions) of their treatment. In addition, there is a need for a thorough and detailed multidisciplinary assessment of the patient presenting with diabetic foot complications. This builds upon the recommendations from previous studies noting that there was significant room for improvement in the assessment of the diabetic foot patient8-9. Lastly, we feel that the availability of a trained nurse counselor to engage and counsel patients in amputation surgery may help in preventing unnecessary delays in patients receiving definitive surgical treatment.
